# Impact of Linker Modification and PEGylation of Vancomycin Conjugates on Structure-Activity Relationships and Pharmacokinetics

**DOI:** 10.3390/ph15020159

**Published:** 2022-01-28

**Authors:** Florian Umstätter, Julia Werner, Leah Zerlin, Eric Mühlberg, Christian Kleist, Karel D. Klika, Tobias Hertlein, Barbro Beijer, Cornelius Domhan, Stefan Zimmermann, Knut Ohlsen, Uwe Haberkorn, Walter Mier, Philipp Uhl

**Affiliations:** 1 Department of Nuclear Medicine, Heidelberg University Hospital, 69120 Heidelberg, Germany; florian.umstaetter@med.uni-heidelberg.de (F.U.); julia.werner@med.uni-heidelberg.de (J.W.); lzerlin96@googlemail.com (L.Z.); eric.muehlberg@web.de (E.M.); christian.kleist@med.uni-heidelberg.de (C.K.); barbro.beijer@med.uni-heidelberg.de (B.B.); uwe.haberkorn@med.uni-heidelberg.de (U.H.); walter.mier@med.uni-heidelberg.de (W.M.); 2 NMR Spectroscopy Analysis Unit, German Cancer Research Center (DKFZ), 69120 Heidelberg, Germany; k.klika@dkfz-heidelberg.de; 3 Institute for Molecular Infection Biology, University of Würzburg, 97080 Würzburg, Germany; tobias.hertlein@uni-wuerzburg.de (T.H.); knut.ohlsen@uni-wuerzburg.de (K.O.); 4 Institute of Pharmacy and Molecular Biotechnology, Heidelberg University, 69120 Heidelberg, Germany; Domhan@uni-heidelberg.de; 5 Department of Infectious Diseases, Medical Microbiology and Hygiene, Heidelberg University Hospital, 69120 Heidelberg, Germany; stefan.zimmermann@med.uni-heidelberg.de; 6 Clinical Cooperation Unit Nuclear Medicine, German Cancer Research Center, 69120 Heidelberg, Germany

**Keywords:** glycopeptide antibiotics, antimicrobial resistance, vancomycin, polycationic peptides, linker influence, pharmacokinetics, PEGylation

## Abstract

As multidrug-resistant bacteria represent a concerning burden, experts insist on the need for a dramatic rethinking on antibiotic use and development in order to avoid a post-antibiotic era. New and rapidly developable strategies for antimicrobial substances, in particular substances highly potent against multidrug-resistant bacteria, are urgently required. Some of the treatment options currently available for multidrug-resistant bacteria are considerably limited by side effects and unfavorable pharmacokinetics. The glycopeptide vancomycin is considered an antibiotic of last resort. Its use is challenged by bacterial strains exhibiting various types of resistance. Therefore, in this study, highly active polycationic peptide-vancomycin conjugates with varying linker characteristics or the addition of PEG moieties were synthesized to optimize pharmacokinetics while retaining or even increasing antimicrobial activity in comparison to vancomycin. The antimicrobial activity of the novel conjugates was determined by microdilution assays on susceptible and vancomycin-resistant bacterial strains. VAN1 and VAN2, the most promising linker-modified derivatives, were further characterized in vivo with molecular imaging and biodistribution studies in rodents, showing that the linker moiety influences both antimicrobial activity and pharmacokinetics. Encouragingly, VAN2 was able to undercut the resistance breakpoint in microdilution assays on *vanB* and *vanC* vancomycin-resistant enterococci. Out of all PEGylated derivatives, VAN:PEG1 and VAN:PEG3 were able to overcome *vanC* resistance. Biodistribution studies of the novel derivatives revealed significant changes in pharmacokinetics when compared with vancomycin. In conclusion, linker modification of vancomycin-polycationic peptide conjugates represents a promising strategy for the modulation of pharmacokinetic behavior while providing potent antimicrobial activity.

## 1. Introduction

Multidrug-resistant bacteria have become an unpredictable burden. By 2050, worldwide, up to 10 million deaths might be caused by bacterial pathogens [1]. Therefore, the development of effective antimicrobial substances should be expedited to counter this threat [2]. However, non-academic research stagnates, and most pharmaceutical companies have abandoned antibiotic development [3,4]. Fortunately, some encouraging developments have been made recently in the academic sector. Due to the immense financial costs of de novo developments, modification of established substances has become a key strategy. In this context, several studies and reviews describe different approaches of modification of one of the most important last-resort antibiotics treating Gram-positive bacterial infections: the glycopeptide antibiotic vancomycin [5,6,7,8,9,10]. Recently, we published a study utilizing a peptide-based modification strategy of vancomycin [11]. The derivatives in this previous study consisted of vancomycin as the core structure, with a polycationic peptide attached via a heterobifunctional linker. The most potent conjugates were shown to overcome all common types of vancomycin resistance, viz., *vanA*, *vanB* and *vanC*. In addition, this modification strategy enabled significant changes in pharmacokinetic behavior when compared to vancomycin. While our previous work was focused on the optimization of antimicrobial activity by addressing different modification sites of vancomycin and on the coupling of various peptide sequences [11], in the present study, optimization of the linker moiety was attempted.

Lessons learned from other compounds, such as antibody-drug conjugates, demonstrate that the linker moiety greatly influences structure–activity relationships and, even more so, pharmacokinetics. Therefore, we investigated the structure-activity relationships of the linker moiety in this study. In general, linkers can be classified with respect to their functional properties. As a primary distinction, crosslinkers can be divided into homobifunctional or heterobifunctional subclasses. Heterobifunctional crosslinkers benefit from a lower rate of unintended cross reactions and are therefore preferred. The most important aspect of crosslinked constructs is their in vivo stability. However, it must be ensured that the crosslinked construct is stable in the bloodstream, as well as in organ/tissue accumulation, to avoid the release of vancomycin. It is therefore imperative to use a linker that provides high in vivo stability. In our case, for example, the peptide moiety used for modification shows no synergistic antimicrobial activity without conjugation to vancomycin [11]. 

**Figure 1:**
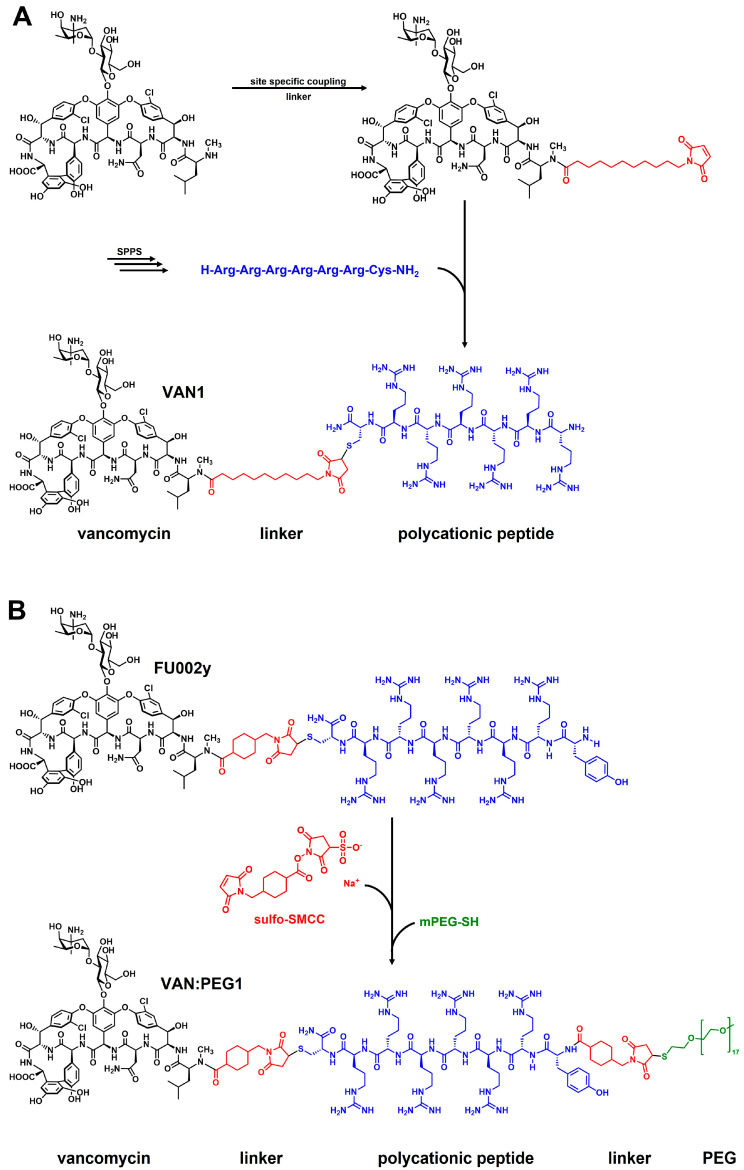
Reaction scheme of the strategies applied. (**A**) Example shown for VAN1 (KMUS linker). In the first step, the bifunctional linker is coupled via the NHS-ester to the secondary amine of vancomycin. In the following step, the hexa-arginine peptide obtained by solid-phase peptide synthesis (SPPS) is coupled to the maleimido group via Michael addition. (**B**) The PEGylation strategy starts from FU002 (in this case, with an additional d-tyrosine for radiolabeling). In the first step, the bifunctional linker SMCC is coupled at the *N*-terminus of the peptide moiety. In the second step, the PEG moiety is coupled via its thiol group, resulting in a stable thioether bond.

Consequently, three different linkers were chosen: *N*-κ-maleimidoundecanoyl-oxysulfosuccinimide ester (KMUS), *N*-α-maleimidoacet-oxysuccinimide ester (AMAS) and a PEGylated, long-chain 4-(N-maleimidomethyl)cyclohexane-1-carboxylate (SMCC) crosslinker (SM(PEG)_12_). All three linkers are heterobifunctional, non-cleavable linkers, and they differ in spacer-arm length. The conjugates were synthesized based on a previously published strategy [11]. In the first step, the conjugation of the linker to vancomycin was performed using the NHS-ester moiety of the linker to address the secondary amine function of vancomycin. Afterwards, a hexa-arginine peptide moiety was coupled to the maleimide unit of the linker in the vancomycin conjugate via a thioether bond. The strategy of the synthesis is outlined in Figure 1A. Microdilution assays on susceptible, as well as vancomycin-resistant, strains were performed to investigate the antimicrobial potential of the novel conjugates in comparison to vancomycin and the previously published vancomycin conjugate FU002 [11].

In a second attempt, in order to modulate the pharmacokinetics, the original highly potent vancomycin conjugate FU002 was PEGylated. Polyethylene glycol (PEG) is often used to enhance the stability of proteins in order to protect against proteolytic digestion, prolong the half-life and many further applications [12,13,14,15,16]. As the original pharmacokinetic behavior of FU002 leads to rapid liver accumulation, PEGylation was used to prolong the half-life in the bloodstream. However, structural modification of an antibiotic comes with the risk of reducing the antimicrobial activity of the compound. To evaluate antimicrobial activity and pharmacokinetic behavior, PEG derivatives of different lengths were added at the *N*-terminus of the conjugates’ peptide moiety via an additional SMCC linker. The syntheses were accomplished by coupling the PEG moiety to the thiol group of the PEG by Michael addition (Figure 1B).

## 2. Results

### 2.1. Conjugate Synthesis and Verification of the Site of Conjugation

Vancomycin conjugates differing in linker moiety (spacer length) were synthesized (Table 1) following the strategy illustrated in Figure 1A. All conjugates were obtained with high purity, as demonstrated by LC/MS analysis (see Supplementary Material). Two coupling sites of vancomycin can be potentially accessed. In the previous study [11], the secondary amine of vancomycin was found to be the preferred modification site for antimicrobial activity; therefore, all conjugates were coupled at this position in the present study.

Due to the applied coupling method, it is possible that the primary amino group of the vancomycin glycoside could also be modified. As previously reported, deglycosylation is a method to verify the modification site [11]. Therefore, in order to verify the coupling position, VAN1 was representatively deglycosylated. LC/MS analysis after deglycosylation clearly proved the coupling position at the secondary amine of the vancomycin core. The calculated molar mass of deglycosylated VAN1 is 2626.72 g/mol. For comparison, the molecular weight of the peptide-linker-sugar moiety is 1480 g/mol (one sugar unit retained) and 1650 g/mol (both sugar units retained). Since a mass of *m*/*z* = 876.71 was observed for a [M + 3H]^3+^ molecular ion, this equates to a mass of 2630 Da (see Supplementary Materials). This unambiguously proves that the peptide moiety was linked to the isoleucine residue, the V_N_ position of the vancomycin core.

For PEGylation, all conjugates were synthesized following the strategy illustrated in Figure 1B, with different PEG-chain lengths (Table 2), and analyzed by MALDI-TOF MS and HPLC.

**Figure 2:**
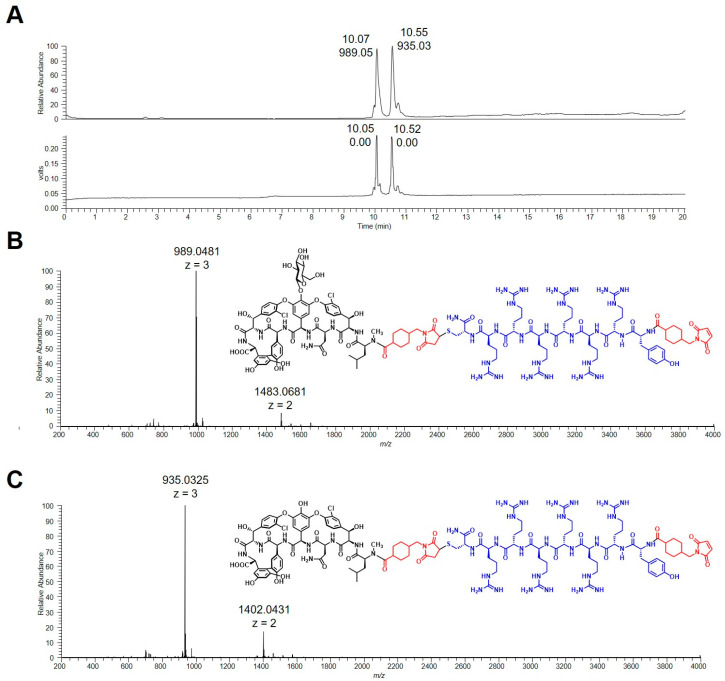
MS and chemical structures of the deglycosylation products of FU002y-SMCC. (**A**) The chromatogram shows two peaks with different masses, indicating two deglycosylated products of FU002y-SMCC. (**B**) The observed mass, *m*/*z* = 989.05 [M + 3H]^3+^, shown in the mass spectrum correlates with the calculated mass of FU002y-SMCC lacking one sugar moiety (2965.04 g/mol). (**C**) The observed mass, *m*/*z* = 935.03 [M + 3H]^3+^, shown in the mass spectrum corresponds to the calculated mass of FU002y-SMCC lacking both sugar moieties (2802.90 g/mol).

In order to confirm the anticipated coupling position, the FU002y-SMCC intermediate was also deglycosylated and analyzed by LC/MS (Figure 2). Moreover, the compound was also examined by NMR to verify the second SMCC coupling at the *N*-terminus of the peptide moiety (see Supplementary Material).

### 2.2. Antimicrobial Activity Testing of Polycationic Peptide-Vancomycin Derivatives

**Figure 3:**
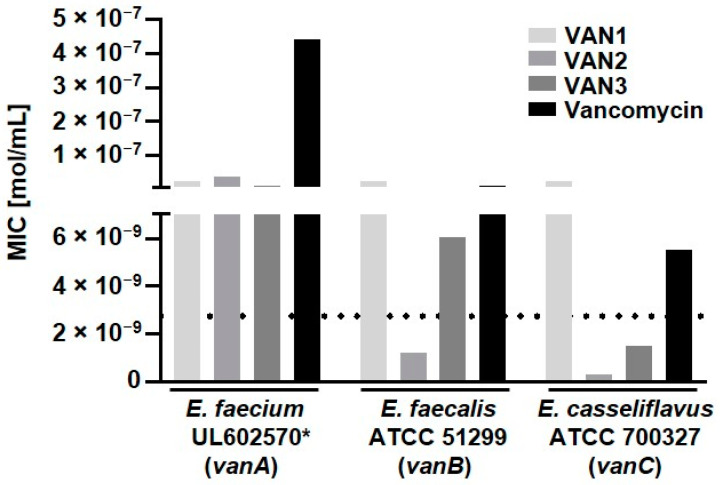
Comparison of the antibiotic activity of the different linker-modified vancomycin-peptide conjugates on vancomycin-resistant bacterial strains. VAN2 was able to overcome the molar-resistance breakpoint for resistant strains of the *vanB* and *vanC* types, while VAN3 also overcame the low-level-resistant *vanC* type. The dotted line represents the level of resistance (EUCAST). (* = clinical isolate).

The conjugates were tested in microdilution assays according to CLSI (Clinical and Laboratory Standards Institute) and EUCAST (European Committee on Antimicrobial Susceptibility Testing) guidelines on both vancomycin-sensitive and-resistant Gram-positive bacterial strains [17,18,19]. All conjugates showed high activity towards susceptible Gram-positive strains (Table S1). More importantly, VAN2, the derivative with the PEG linker, and VAN3, the compound containing the shortest linker, were able to overcome the molar-resistance breakpoint in microdilution assays for at least one of the vancomycin-resistant bacteria (Figure 3).

**Figure 4:**
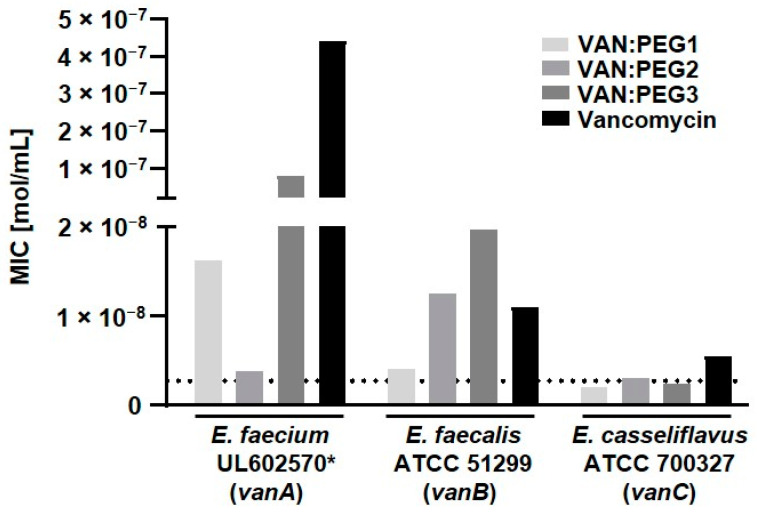
Comparison of the antibiotic activity of the PEGylated vancomycin-peptide conjugates on vancomycin-resistant bacterial strains. VAN:PEG1 and VAN:PEG3 are able to overcome the molar-resistance breakpoint on resistant bacteria of the *vanC* type. The dotted line represents the level of resistance (EUCAST). (* = clinical isolate).

After PEGylation, the second modification approach, all derivatives clearly showed a loss of effectiveness against the tested antimicrobial strains in comparison to the originator, FU002 (Figure 4) (data for FU002 published previously [11]). These findings clearly indicate the strong influence of the linker structure-activity relationship on the vancomycin-peptide conjugates, as well as the influence of PEGylation.

### 2.3. Hemolysis Studies

To determine the toxicity to red blood cells and thus confirm the selectivity to bacteria, a hemoglobin release assay was carried out with VAN1, VAN2 and VAN3, as well as the PEGylated conjugate, VAN:PEG1. None of the compounds showed significant hemolytic activity in concentrations far above the determined MIC (Tables S2 and S3).

### 2.4. Molecular Imaging and Biodistribution Studies

**Figure 5:**
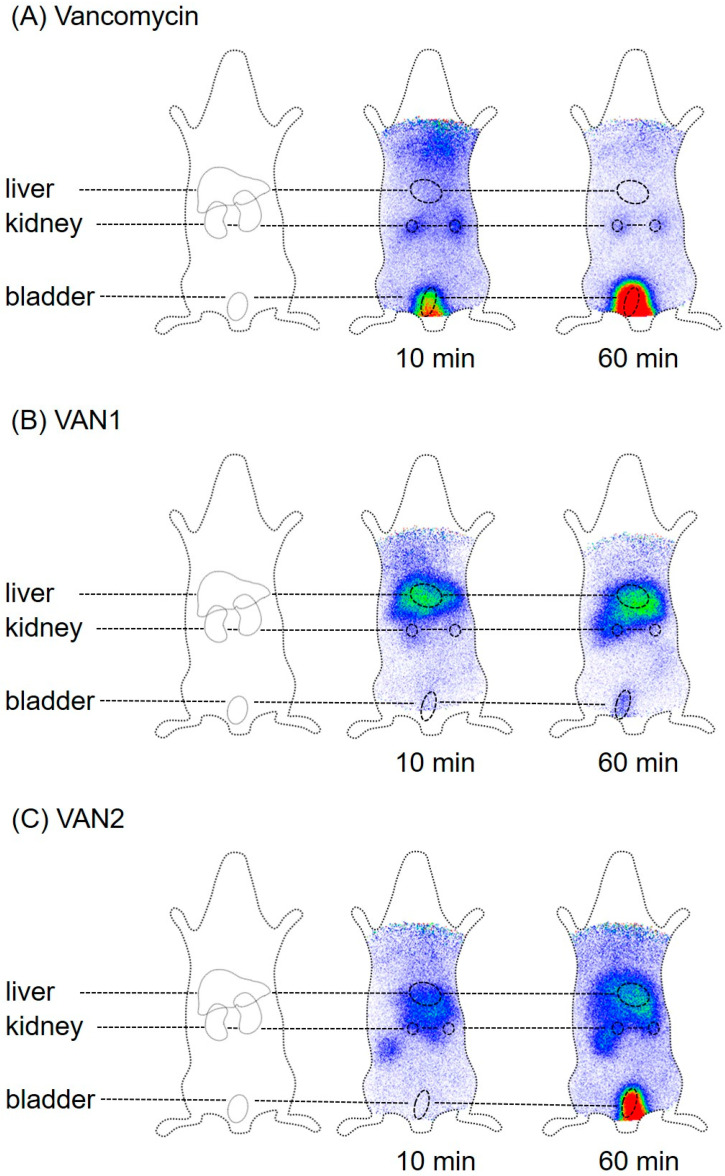
Comparison of the pharmacokinetic behavior of vancomycin and novel vancomycin-peptide conjugates VAN1 and VAN2 by molecular imaging. (**A**) Vancomycin shows a fast renal elimination. (**B**) By contrast, VAN1 accumulates quickly in the liver, as previously shown for FU002 [11]. (**C**) VAN2 also exhibits modified pharmacokinetics. Although the behavior of VAN2 differs from that of VAN1, rapid liver accumulation can still be observed, in addition to renal elimination.

Molecular imaging and biodistribution studies were performed for VAN1 and VAN2, as well as for VAN:PEG1 and VAN:PEG2, using female Wistar rats. Both VAN1 and VAN2 showed an altered biodistribution profile in comparison to vancomycin. The conjugates VAN1 and VAN2 target mainly the liver, in comparison to vancomycin, which shows a renal excretion profile (Figure 5). These findings clearly demonstrate the ability to modulate pharmacokinetics by means of the presented modification methods.

**Figure 6:**
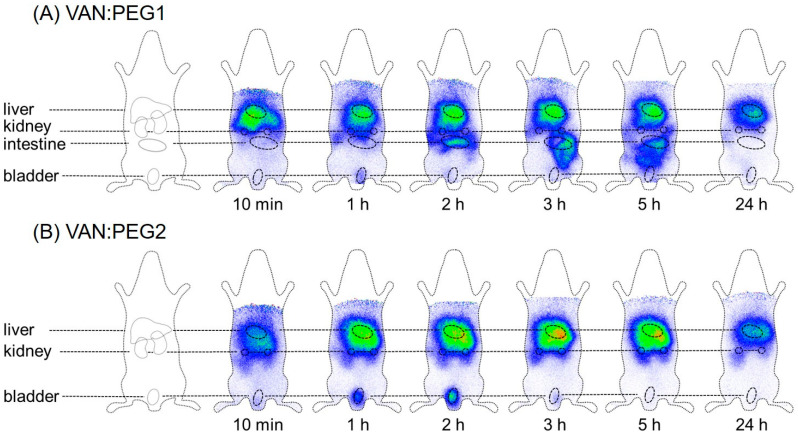
Comparison of the pharmacokinetic behavior of VAN:PEG1 and VAN:PEG2 by molecular imaging. (**A**) VAN:PEG1 shows significantly changed pharmacokinetic behavior as it accumulates in the liver and is predominantly excreted by the intestine. (**B**) In contrast, VAN:PEG2 accumulates rapidly in the liver, as shown previously for FU002 [11].

The PEGylated conjugates VAN:PEG1 and VAN:PEG2 also exhibited significantly different biodistribution behavior in molecular imaging studies (Figure 6).

To further compare the two modification strategies, the biodistribution profiles of VAN1, VAN2 and vancomycin were compared using excised organs. The different organ-tissue/blood ratios are shown in Table 3. This study confirms significant liver accumulation in most cases.

## 3. Discussion

Highly active antimicrobials to serve as treatment options for multidrug-resistant bacteria are urgently required [20,21]. However, antimicrobial activity is not the only requirement for a compound to be suitable for clinical application [22]. Frequently, the potential of active substances cannot be exploited due to adverse reactions, e.g., nephrotoxicity, as exemplified by vancomycin itself [23,24]. Moreover, methods of modulation of the biodistribution profile, as well as pharmacokinetics, should be considered as important as the antimicrobial potency of novel compounds [25]. With respect to structural modification of vancomycin, strategies for optimization of its pharmacokinetics and toxicity, accompanied by high antimicrobial activity, were previously published [26]. For our lead compound. FU002 [11], its short plasma half-life limits its further development. For this reason, in the current study, the influence of linker characteristics was investigated with respect to blood circulation and biodistribution behavior.

The sophisticated modification strategy used in this study is based on established bioconjugation strategies, and we were able to successfully transfer the modification process to chemically related linkers varying in chain length. Owing to similar functionalities, these linkers could be coupled, as previously established for FU002 [11]. Although the synthesized substances showed activity in microdilution assays, not all derivatives showed the ability to overcome the resistance breakpoint of vancomycin in vancomycin-resistant strains. These findings clearly demonstrate the importance of spacer length between vancomycin and its polycationic peptide modification for retaining antimicrobial efficacy, as well as the fact that a defined spatial structure is needed to maintain antimicrobial activity. Due to their significant antimicrobial potential in comparison to vancomycin, several compounds were further characterized in vivo by molecular imaging and biodistribution studies. In these studies, as expected based on the altered linker characteristics, VAN1 and VAN2 showed a remarkable change in pharmacokinetic behavior compared to vancomycin, as demonstrated by different organ distributions. Additionally, due to the modified linker moiety, pharmacokinetics differed between VAN1 and VAN2. VAN2, containing the PEG linker moiety, is more hydrophilic, thereby resulting in greater renal elimination. These findings clearly indicate the strong influence of the linker moiety and spacer length for in vivo characteristics, and we therefore strongly recommend taking these findings into account for future reactivation strategies of established compounds.

Vancomycin itself is known for its rapid and almost complete renal elimination, which is associated with nephrotoxicity in high doses, especially in multimorbid patients. Reduced renal elimination results in higher vancomycin blood levels [27]. Considering the biodistribution profiles of the novel conjugates, we hypothesize that this limitation could be bypassed by the primary hepatobiliary elimination route. The PEGylation strategy also resulted in changed pharmacokinetics, even in comparison to FU002. Unfortunately, antimicrobial activity decreased with this modification. Although the mode of action of the novel conjugates remains unclear, the observation of the current study that steric hindrance leads to a weakening of the antimicrobial effect supports the hypothesis that the modified derivatives bind to a defined target structure [11]. Besides the partial loss of antimicrobial activity in comparison to our lead compound, FU002, a further limitation seems to be the poor yield obtained in chemical synthesis. The required coupling of the additional second SMCC linker enabling the PEGylation complicates the synthesis strategy and therefore reduces the yield of synthesis.

These results demonstrate the linker structure-activity relationship and the impact on pharmacokinetics. For these presented modification strategies, it has to be considered that a suitable linker that addresses the pharmacokinetics does not automatically provide enhancement of antimicrobial activity. Thus, the choice of optimal linker requires selection on a case-by-case basis. However, the aim of modulating the pharmacokinetic properties of antibiotic-peptide conjugates was not achieved in a satisfactory manner. Therefore, other strategies, such as the use of nanocarriers, should be investigated in subsequent studies. It is well known in the literature that modification of the liposomal surface represents a versatile strategy for modulation of pharmacokinetic behavior and to enable specific organ targeting [28]. Prolongation of plasma half-life can be achieved by PEGylation of nanocarriers [29]. The encapsulation of the new vancomycin derivatives in PEGylated liposomes, for example, is therefore a preferred strategy to be tested in future studies.

## 4. Materials and Methods

### 4.1. General

Fmoc-l-amino acids were purchased for synthesis of the polycationic peptides from Orpegen Peptide Chemicals GmbH, Heidelberg, Germany. Fmoc-d-tyrosine and rink amide resin were obtained from Iris Biotech GmbH, Marktredwitz, Germany. Vancomycin hydrochloride for synthesis of the conjugates was received from Noridem Enterprises Limited, Hallbergmoos, Germany and Hikma Pharma GmbH, Planegg, Germany. *N*-κ-Maleimidoundecanoyl-oxysulfosuccinimide ester was obtained from TCI Deutschland GmbH, Eschborn, Germany, and *N*-α-maleimidoacet-oxysuccinimide ester was obtained from ABCR GmbH, Karlsruhe, Germany. mPEG-SH (average M_n_, 800 Da; average M_n_, 2000 Da); vancomycin hydrochloride (potency 99.8%), serving as a control in antimicrobial activity assays; Mueller-Hinton-Broth II (cation-adjusted) and *O*-[2-(3-mercaptopropionylamino)ethyl]-*O′*-methylpolyethylene glycol (average M_n_, 5000 Da) were obtained from Sigma-Aldrich, Merck KGaA, Darmstadt, Germany. All purification steps were performed by preparative HPLC using a LaPrep P 110 (VWR International, Darmstadt, Germany) HPLC system equipped with a Reprosil™ Gold 120 C-18 column (4 μm, 150 × 20 mm). Analyses were performed by LC/MS using an Exactive Orbitrap Mass Spectrometer, Thermo Fisher Scientific, Dreieich, Germany. For microdilution assays, the cell number was adjusted using a McFarland-counter DensiCHEK^®^ plus, Biomerieux, Marcy-l’Étoile, France. For determination of antimicrobial activity, u-bottom 96-well polypropylene plates were purchased from Greiner Bio-One International GmbH, Kremsmünster, Austria. All clinical isolates were obtained from the Institute for Medical Microbiology and Hygiene, Heidelberg University Hospital, Heidelberg, Germany. The ATCC reference strains were obtained from LGC Standards GmbH, Wesel, Germany. Rats for biodistribution studies and molecular imaging were supplied by Janvier labs, Le Genest-Saint Isle, France. ^125^I was purchased from Hartmann Analytic, Braunschweig, Germany. For scintigraphic imaging, a γ-camera (Gamma Imager, Biospace, France) was used. For the biodistribution studies, the activity in the organs was measured using a Cobra Auto γ-Counter, Packard Bioscience, USA.

### 4.2. Experimental Section

#### 4.2.1. Chromatographic Analytics

Analyses of intermediate and final products were performed by HPLC using a linear gradient of 0.1% trifluoroacetic acid (TFA) in water (eluent A) to 0.1% TFA in acetonitrile (eluent B) within 5 min (flow rate, 2 mL/min; UV absorbance, λ = 214 nm) and by LC/MS using a gradient of water and acetonitrile, both containing 0.05% TFA and, with a flow rate of 200 μL/min for HPLC.

#### 4.2.2. Peptide Synthesis

The polycationic peptide, consisting of six arginines and one C-terminally located cysteine, was synthesized by solid-phase peptide synthesis using the Fmoc strategy. Briefly, a rink amide resin (loading 0.67 mmol/g) was used for standard coupling strategy using an Applied Biosystems 433A synthesizer with HBTU activation. After the final Fmoc deprotection and washing, the resin was additionally washed with diethyl ether and dried under vacuum. The final cleavage of the peptide was performed in TFA/H_2_O/TIS (95/2.5/2.5) for a minimum of 2 h. Precipitation of the cleaved peptide was performed in diethyl ether. Purification of the product was performed by preparative HPLC.

#### 4.2.3. Synthesis of the Conjugates

For synthesis of the novel conjugates, the previously described strategy was used [11]. For this purpose, vancomycin hydrochloride was dissolved in standard phosphate-buffered saline (PBS) (pH = 8.16) and mixed with an equimolar DMSO stock solution of the respective linker (KMUS for VAN1, SM(PEG)_12_ for VAN2 and AMAS for VAN3). The mixture was shaken at room temperature, and the reaction was monitored by HPLC. Purification was performed by preparative HPLC. The resulting product was analyzed by LC/MS and lyophilized for further processing. The resulting vancomycin-linker conjugate was then dissolved in PBS (pH = 5.4) and DMSO (1:1 *v*/*v*) and mixed in PBS (pH = 5.4) with an equimolar amount of peptide. The reaction was monitored by HPLC, and upon completion of the reaction, purification and analysis were performed as described above, and the final product was lyophilized. For PEGylated derivatives, the previously published tyrosine-modified derivative of FU002 (FU002y) was synthesized. To enable PEGylation with methoxy PEG thiol (mPEG-SH), an additional SMCC linker was coupled to the *N*-terminus of the peptide moiety of FU002y. For this purpose, FU002y was mixed with a solution of SMCC in DMSO at pH = 8.16. This mixture was incubated at room temperature for 30 min and monitored by HPLC. After completion of the reaction, the intermediate product, FU002y-SMCC, was purified by preparative HPLC and analyzed by LC/MS. For PEGylation, this intermediate was mixed at pH = 5.4 with 0.5 eq of a mPEG-SH in DMSO stock (average weights 800 Da (VAN:PEG1), 2000 Da (VAN:PEG2) and 5000 Da (VAN:PEG3)). The final products were purified by preparative HPLC. For analysis of PEGylated FU002y, a MALDI-TOF MS instrument (Bruker Microflex LT, Ettlingen, Germany) was used.

#### 4.2.4. Structural Analysis by Deglycosylation

Two amine functionalities of vancomycin could possibly participate in the reactions. To exclude the undesired primary amine functionality of the sugar moiety of vancomycin as the site of conjugation and thus confirm the secondary amine of the vancomycin core as the site of conjugation, and similarly for the *N*-terminus of the peptide moiety in the case of PEGylation, deglycosylation was performed. VAN1 or FU002y-SMCC was dissolved in water and incubated with TFA. After 12 to 18 h, the TFA and water were removed under vacuum, and the solid residue was dissolved in water/acetonitrile (1:1). Analysis was performed by LC/MS, as described above. 

#### 4.2.5. NMR

NMR spectra were acquired using a Bruker Avance II NMR spectrometer equipped with a 5-mm inverse-configuration probe with triple-axis gradient capability at a field strength of 14.1 T, operating at 600 MHz for ^1^H nuclei in *δ*_6_-DMSO at 298 K. Pulse widths were calibrated following the described protocol [30]. The chemical shifts of ^1^H nuclei are reported in ppm relative to TMS (*δ* = 0 ppm), using the solvent signal as a secondary internal reference (*δ*_CHD2SOCD3_ = 2.50 ppm), while coupling constants (*J*) are given in Hz. General NMR experimental and acquisition details for 1D ^1^H and selective 1D NOESY (*τ*_m_, 0.3 s) and standard, gradient-selected 2D COSY and ^1^H–^13^C HSQC have been previously described [31,32,33,34] for routine spectral assignment and structural analysis. Legend: br, broad; d, doublet; dAB, doublet with higher-order character; *J*_L_, *ortho* coupling in AA’XX’ multiplet; *J*_S_, *para* coupling in AA’XX’ multiplet; m, multiplet; ol, overlapped; qt, quartet; t, triplet; s, singlet; v, very.

#### 4.2.6. Antimicrobial Testing: Microdilution

Antimicrobial activity assays were performed by microdilution and conducted in accordance with CLSI and EUCAST guidelines [17,18,19]. Serial dilutions in MHB II (cation adjusted) were performed on 96-well plates using water-stock solutions of the compounds at different concentrations. Overnight cultures of bacterial strains were used to obtain bacteria in the logarithmic growth phase. Bacteria were adjusted to a McFarland corresponding to 1 × 10^8^ cfu/mL. After a 1:100 dilution, the culture broth was loaded onto the 96-well polypropylene plates, resulting in a final bacterial concentration of 5 × 10^5^ cfu/mL. Plates were then incubated at 37 ± 1 °C for 18–24 h. The MICs were assessed as the lowest concentration without visible growth.

#### 4.2.7. Hemoglobin Release Assay

To exclude lytic activity and to evaluate the toxicological potential of the conjugates, hemolysis studies were performed as previously described [11]. Briefly, fresh heparinized blood of four male, fasted volunteers was taken, and erythrocytes were purified by centrifugation of the blood at 2500 rpm for 10 min. The supernatant was removed, and the resulting pellet was washed with PBS at least three times, until the supernatant remained clear. The conjugates were serially diluted from stock solutions (in phosphate-buffered saline) in a 96-well plate at concentrations ranging from 120 µg/mL to 0.23 µg/mL (VAN1) or 16 µg/mL to 0.03 µg/mL (VAN2, VAN3 and VAN:PEG1), resulting in a final volume of 50 µL. A solution of Triton^®^X-100 in PBS was used as positive control. Finally, 50 µL of the washed erythrocytes was loaded into each well. After incubation for 30 min at 37 °C, 75 µL PBS was added to each well, and the plates were centrifuged for 5 min at 4000 rpm. A total of 50 µL of the supernatants was then transferred into a new polystyrene, flat-bottom 96-well plate, and the absorbances were measured at 554 nm using a Tecan microplate reader. Hemolysis was calculated as follows:

$$Hemolysis\;\left[\%\right]=100\times \frac{A_{substance}-A_{neg.\;control}}{\begin{array}{c} A_{Triton}-A_{neg.\;control} \end{array}}$$

The negative control was erythrocytes, treated as described but using only PBS.

#### 4.2.8. Biodistribution and Pharmacokinetic Studies

Ethical statement: The animal studies included in this manuscript were approved by the Animal Care and Use committees at the Regierungspräsidium Karlsruhe, Germany (reference number 35-9185.81/G-111/16; date of approval: 22 June 2016).

For biodistribution and pharmacokinetic studies, compounds were radiolabeled with ^125^I. The previously described method for the insertion of an additional d-tyrosine to the *N*-terminus of the peptide moiety was used to enable nucleophilic substitution for VAN1 and VAN2 [11]. For the PEGylated derivatives, VAN:PEG1 and VAN:PEG2, tyrosine-modified FU002y was used to synthesize the intermediate. Radiolabeling was performed using the chloramine T method as previously described [35]. Thus, a 1 mM stock solution of the respective compound in phosphate buffer/dimethyl sulfoxide was prepared. The required amount of the radionuclide (^125^I) was added to a mixture of 25 μL of the stock solution and 25 μL of phosphate buffer (0.25 mM; pH = 7.5). Purification of the labeled compounds was performed by semi-preparative HPLC. Analysis was conducted by radio HPLC (Agilent 1100 series) using a Chromolith^®^ Performance RP-18e, 100 × 3 mm column, applying a linear gradient of 0.1% TFA in water (eluent A) to 0.1% TFA in acetonitrile (eluent B) within 5 min (flow rate, 2 mL/min; UV absorbance, λ = 214 nm; γ-detection). Animals were anaesthetized by isoflurane inhalation, and appropriate amounts of the radiolabeled conjugate dissolved in 100 µL 0.9% NaCl solution were injected into the tail vein. Cumulative scintigraphic images were taken after 10 min and 1, 2, 3, 5 and 24 h. For the biodistribution studies, animals were sacrificed at the indicated points in time. The activity of the weighed organs was measured in comparison with standards. Tissue-associated activity was related to the total injected dose (ID) and expressed as a percentage of the total injected dose per gram of tissue (%ID/g). The data were calculated afterwards to obtain the respective organ/organ or organ/blood ratios.

## 5. Conclusions

In this study, we demonstrated the significant influence of linker characteristics on both the antimicrobial potential and the pharmacokinetics of vancomycin conjugates. Although pharmacokinetic behavior could be modulated by changing the linker moiety of the conjugates, as well as by the PEGylation of the previously described FU002, even slight modifications could result in a significant decrease in antimicrobial activity. Nevertheless, the conjugates were able to enhance activity against vancomycin-resistant bacteria in comparison to vancomycin. Based on the absence of hemolytic activity, it was possible to design conjugates with encouraging properties. Thus, the linker structure-activity relationship and its impact on pharmacokinetics should also be investigated for other vancomycin conjugates published previously in order to enable the generation of compounds with optimal properties.

## Figures and Tables

**Table 1:** Chemical structure and characteristics of the conjugates in comparison to the previously published compound FU002 [11]. The conjugates differ in spacer-arm length (in red), but all possess the same polycationic hexa-arginine peptide moiety (R6C).

Compound	Structure	Spacer [Å]	[g/mol]
FU002 (SMCC linker)	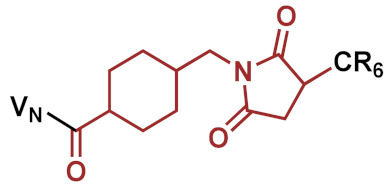	8.3	2725.81
VAN1 (KMUS linker)	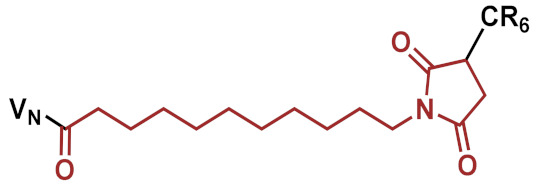	16.3	2769.91
VAN2 (PEG linker)	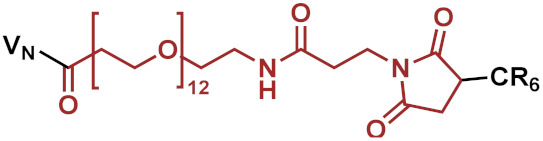	53.4	3257.41
VAN3 (AMAS linker)	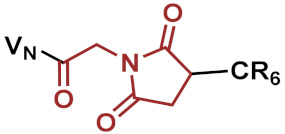	4.4	2643.69

**Table 2:** Overview of the synthesized PEGylated conjugates. The conjugates differ in PEG-chain length, but all possess the same polycationic hexa-arginine peptide moiety (R6C).

Compound	PEG Derivative	Average M_n_ of PEG Unit [g/mol]	Calculated Final Molar Mass [g/mol]
VAN:PEG1	mPEG-thiol	800	3943.3
VAN:PEG2	mPEG-thiol	2000	5094.6
VAN:PEG3	Mercapto-PEG-monomethyl ether	5000	8108.2

**Table 3:** Comparison of vancomycin, VAN1 and VAN2 biodistributions. The organ/blood ratios indicate an increased circulation for VAN2 in comparison to all other tested derivatives. Ratios were measured 10 min post injection.

Compound	Ratio Liver/Kidney	Ratio Liver/Blood	Ratio Kidney/Blood
Vancomycin	0.17	0.36	2.16
VAN1	4.47	10.13	2.22
VAN2	0.92	9.25	10.09

## Data Availability

Data are contained within the article and in the Supplementary Material (Figures S1–S12), NMR studies and NMR data and Tables S1–S3).

## Supplementary Materials

The following supporting information can be downloaded at https://www.mdpi.com/article/10.3390/ph15020159/s1, Figure S1: Mass spectrum and structure of peptide moiety R6C, Figure S2: Mass spectrum and structure of VAN1, Figure S3: Mass spectrum and structure of VAN2, Figure S4: MS spectrum and structure of VAN3, Figure S5: MS and structure of VAN1 after deglycosylation, Figure S6: ^1^H NMR spectrum of SMCC, Figure S7: Downfield region of the ^1^H NMR spectrum of SMCC, Figure S8: Upfield region of the ^1^H NMR spectrum of SMCC, Figure S9: ^1^H NMR spectrum of FU002ySMCC, Figure S10: ^1^H NMR spectrum of FU002ySMCC scaled up, Figure S11: Downfield region of the ^1^H NMR spectrum of FU002ySMCC, Figure S12: Upfield region of the ^1^H NMR spectrum of FU002ySMCC, NMR studies and NMR data, Table S1: Minimum inhibitory concentration against *B. subtilis* DSM10, Table S2: Hemolysis study of VAN1, Table S3: Hemolysis study of VAN2, VAN3 and VAN:PEG1.
