# Supplementary material for: Impact of Linker Modification and PEGylation of Vancomycin Conjugates on Structure-Activity Relationships and Pharmacokinetics
Source: Pharmaceuticals (Basel). 2022 Jan 28;15(2):159. doi: 10.3390/ph15020159 (PMC8880691; doi:10.3390/ph15020159)
Supplement: Supplementary file 1 [file pharmaceuticals-15-00159-s001.zip › pharmaceuticals-1533295-supplementary.pdf]

Supporting information:

Impact of Linker Modification and PEGylation of Vancomycin Conjugates on Structure-Activity Relationships and Pharmacokinetics

Florian Umstätter [1,†], Julia Werner [1,†], Leah Zerlin [1], Eric Mühlberg [1], Christian Kleist [1], Karel D. Klika [2], Tobias Hertlein [3], Barbro Beijer [1], Cornelius Domhan [4], Stefan Zimmermann [5], Knut Ohlsen [3], Uwe Haberkorn [1,6], Walter Mier [1] and Philipp Uhl [1,*]

[1] Department of Nuclear Medicine, Heidelberg University Hospital, 69120 Heidelberg, Germany; florian.umstaetter@med.uni-heidelberg.de (F.U.); julia.werner@med.uni-heidelberg.de (J.W.); lzerlin96@googlemail.com (L.Z.); eric.muehlberg@web.de (E.M.); christian.kleist@med.uni-heidelberg.de (C.K.); barbro.beijer@med.uni-heidelberg.de (B.B.); uwe.haberkorn@med.uni-heidelberg.de (U.H.); walter.mier@med.uni-heidelberg.de (W.M.); philipp.uhl@med.uni-heidelberg.de (P.U.)

[2] NMR Spectroscopy Analysis Unit, German Cancer Research Center (DKFZ), 69120 Heidelberg, Germany; k.klika@dkfz-heidelberg.de

[3] Institute for Molecular Infection Biology, University of Würzburg, 97080 Würzburg, Germany; tobias.hertlein@uni-wuerzburg.de (T.H.); knut.ohlsen@uni-wuerzburg.de (K.O.)

[4] Institute of Pharmacy and Molecular Biotechnology, Heidelberg University, 69120 Heidelberg, Germany; Domhan@uni-heidelberg.de

[5] Department of Infectious Diseases, Medical Microbiology and Hygiene, Heidelberg University Hospital, 69120 Heidelberg, Germany; stefan.zimmermann@med.uni-heidelberg.de

[6] Clinical Cooperation Unit Nuclear Medicine, German Cancer Research Center, 69120 Heidelberg, Germany

* Correspondence: philipp.uhl@med.uni-heidelberg.de; Tel.: +49-6221-567726

† These authors contributed equally to this work.

Characterization of the peptide and conjugates by mass spectrometry

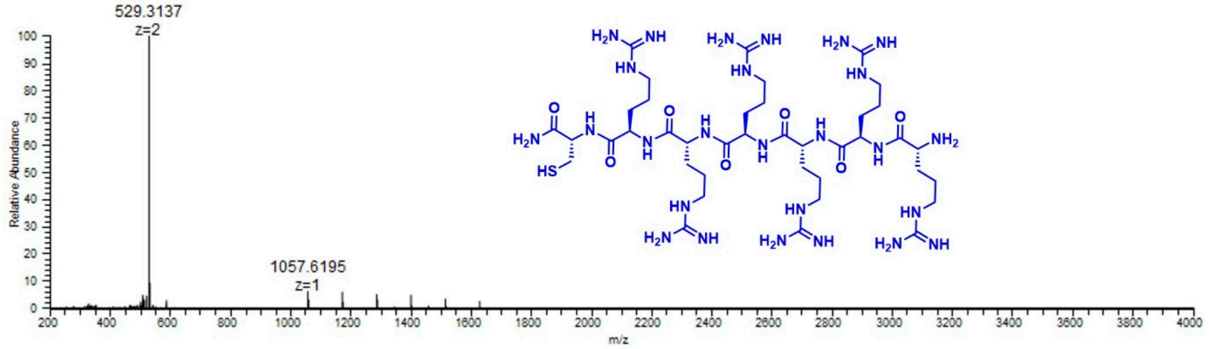

Figure S1. Mass spectrum and structure of the peptide moiety R6C. A mass of m/z = 529.31 $[M + 2H]^{2+}$ was observed and corresponds to the calculated mass of the peptide.

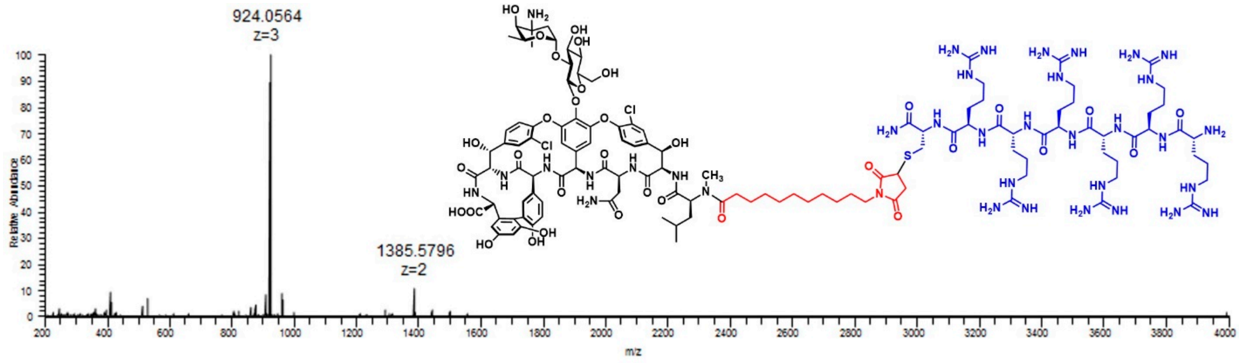

Figure S2: Mass spectrum and structure of VAN1. A mass of m/z = 924.06 $[M + 3H]^{3+}$ was observed and corresponds to the calculated mass of the conjugate.

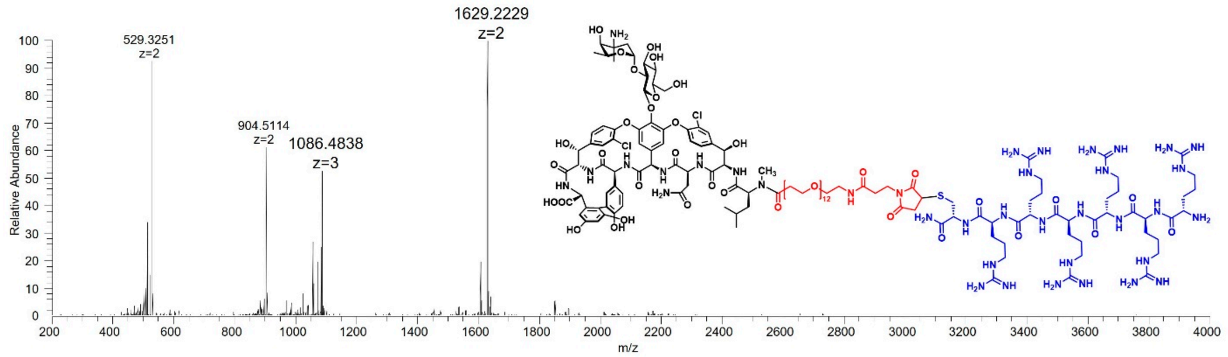

Figure S3. Mass spectrum and structure of VAN2. A mass of m/z = 1629.22 $[M + 2H]^{2+}$ was observed and corresponds to the calculated mass of the conjugate. m/z = 529.33 $[M + 2H]^{2+}$corresponds to the R6C peptide moiety and m/z = 904.51 $[M + 2H]^{2+}$ corresponds to linker-peptide and are mass specific fragments.

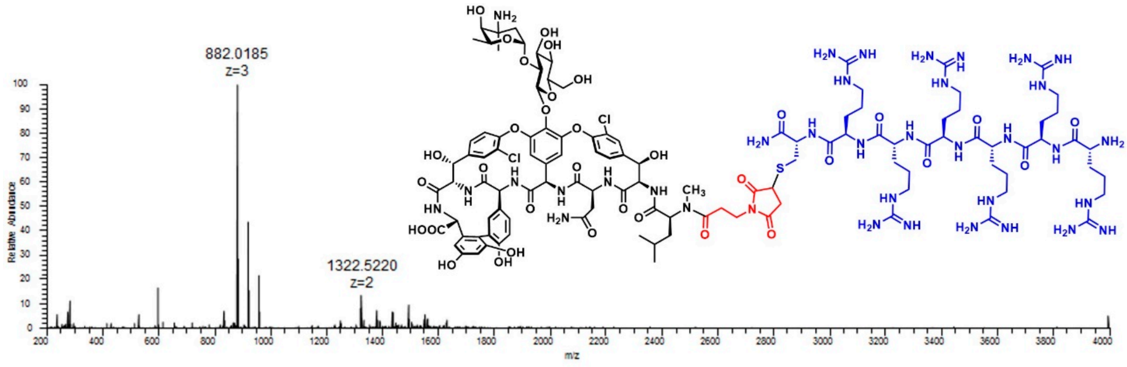

Figure S4: Mass spectrum and structure of VAN3. A mass of m/z = 882.02 $[M + 3H]^{3+}$ was observed and corresponds to the calculated mass of the conjugate.

Structural determination and characterization of VAN1 by deglycosylation

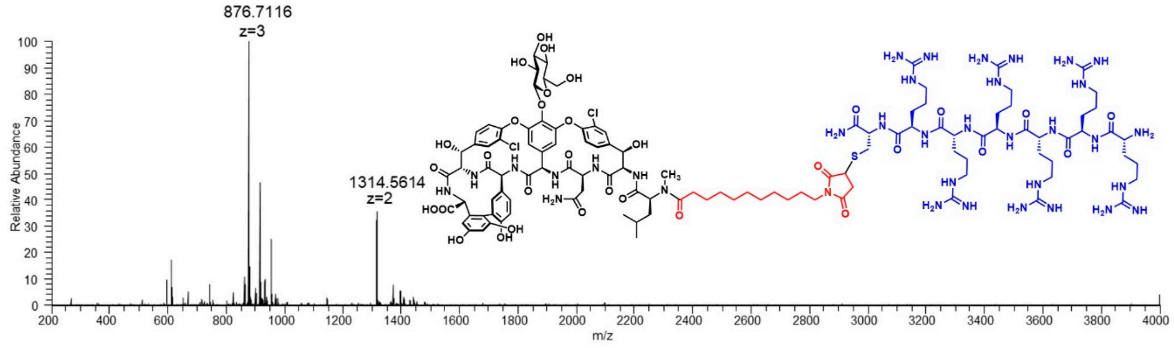

Figure S5. Mass spectrum and structure of VAN1 after deglycosylation. A mass of m/z = 876.71 $[M + 3H]^{3+}$ was observed and proves that the peptide moiety was linked to the isoleucine residue, the V_N position of the vancomycin core.

NMR studies

Structural determination and characterization of the conjugates

The site of attachment of the sidechain in SMCC (succinimidyl 4-(*N*-maleimidomethyl)cyclohexane-1-carboxylate) conjugated to vancomycin was confirmed by 1D NOESY and changes in the chemical shifts in comparison with vancomycin [S1]. The proton and carbon chemical shifts taken from HSQC of the amino sugar in SMCC do not differ from those in vancomycin whereas the shifts in the isobutyl chain are significantly shifted. The proton chemical shifts also differ in a phenyl group that can be only close to the isobutyl chain and not the vancomycin sugar and for which an NOE between protons in the introduced moiety and a proton in that phenyl group can be observed.

The SMCC analysis was the basis for also confirming the site of attachment in FU002y-SMCC of the second SMCC unit whereby the informative proton chemical shifts of the vancomycin sugar were identical to those in SMCC conjugated to vancomycin, thereby indicating that the site of attachment of the second SMCC must lie elsewhere, namely at the terminus of the peptide chain.

NMR data

Legend: br, broad; d, doublet; m, multiplet; ol, overlapped; qt, quartet; t, triplet; s, singlet; v, very.

SMCC (succinimidyl 4-(*N*-maleimidomethyl)cyclohexane-1-carboxylate) conjugated to vancomycin

^{1}H (600 MHz, DMSO): 9.95, s; 9.38, s; 8.78, br d, *J* = 6.0; 8.70, v br d, *J* = 4.0; 7.82, br d, *J* = 1.3; 7.69, v br s; 7.62, 4H, v br s; 7.56, br dd, *J* = 8.4 and 1.3; 7.45, br dd, *J* = 8.4 and 1.3; 7.33, d, *J* = 8.4; 7.20, d, *J* = 8.4; 7.06, br s; 6.93, v br s; 6.90, 2H, s; 6.81, br dd, *J* = 8.4 and 1.8; 6.72, d, *J* = 8.6; 6.68, d, *J* = 2.2; 6.66, br s; 6.56, d, *J* = 2.3; 5.98, br d, *J* = 3.4; 5.94, d, *J* = 6.4; 5.82, d, *J* = 7.7; 5.67, s; 5.47, d, *J* = 6.6; 5.33, d, *J* = 5.8; 5.26, d, *J* = 7.8; 5.24, br d, *J* = 3.8; 5.16, br t, *J* = 3.5; 5.12–5.14, 2H, m; 5.07, d, *J* = 4.7; 4.82, v br d, *J* = 6.3; 4.67, qt, *J* = 6.5; 4.59, d, *J* = 6.3; 4.42, br d, *J* = 5.4; 4.15, br d, *J* = 11.7; 4.09, br s; 4.03, t, *J* = 5.4; 3.71–3.67, m; 3.56–3.51, 2H m; 3.43, ~qt, *J* = 7.8; 3.29–3.25, m; 3.18, br d, *J* = 6.5; 3.05, 2H, br d, *J* = 6.8; 2.72–2.62, br, m; 2.52, 3H, br s; 2.02–1.94, m; 1.93–1.85, m; 1.79–1.33, 8H, m; 1.29, 3H, s; 1.28–1.21, 2H, m; 1.06, 3H, d, *J* = 6.4; ca. 1.03, ol m; 0.93, 3H, d, *J* = 6.5; 0.87, 3H, d, *J* = 6.6; ca. 0.83, ol m; 0.74–0.62, 3H, m.

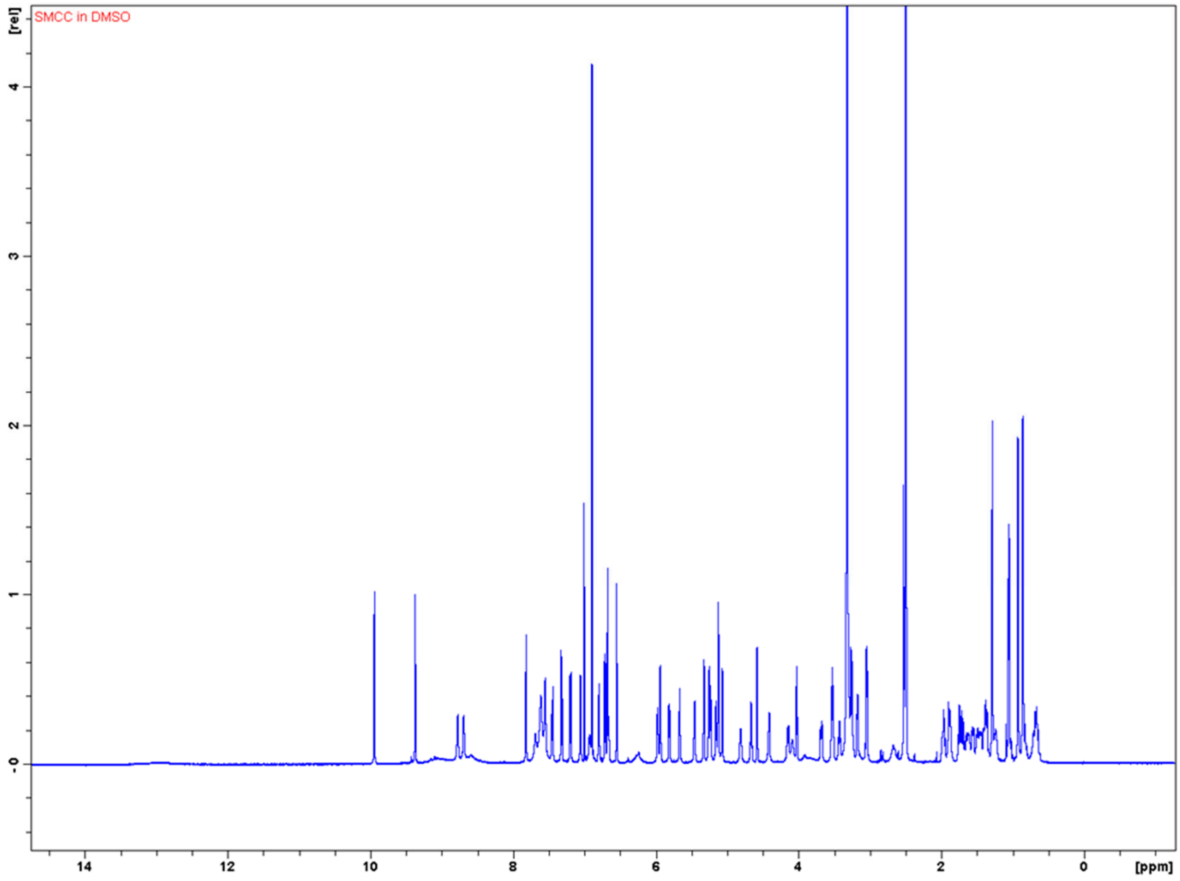

Figure S6. ^{1}H NMR spectrum of SMCC.

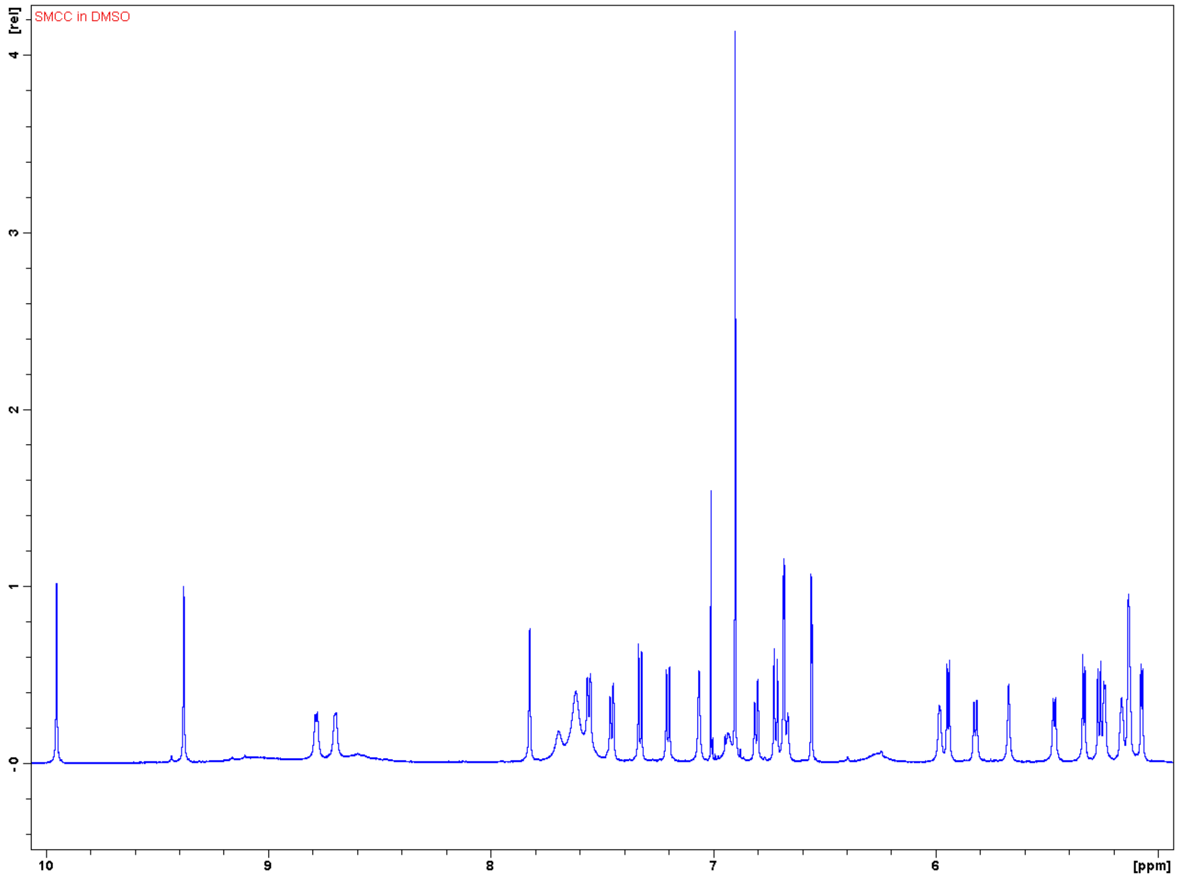

Figure S7. Downfield region of the ^{1}H NMR spectrum of SMCC.

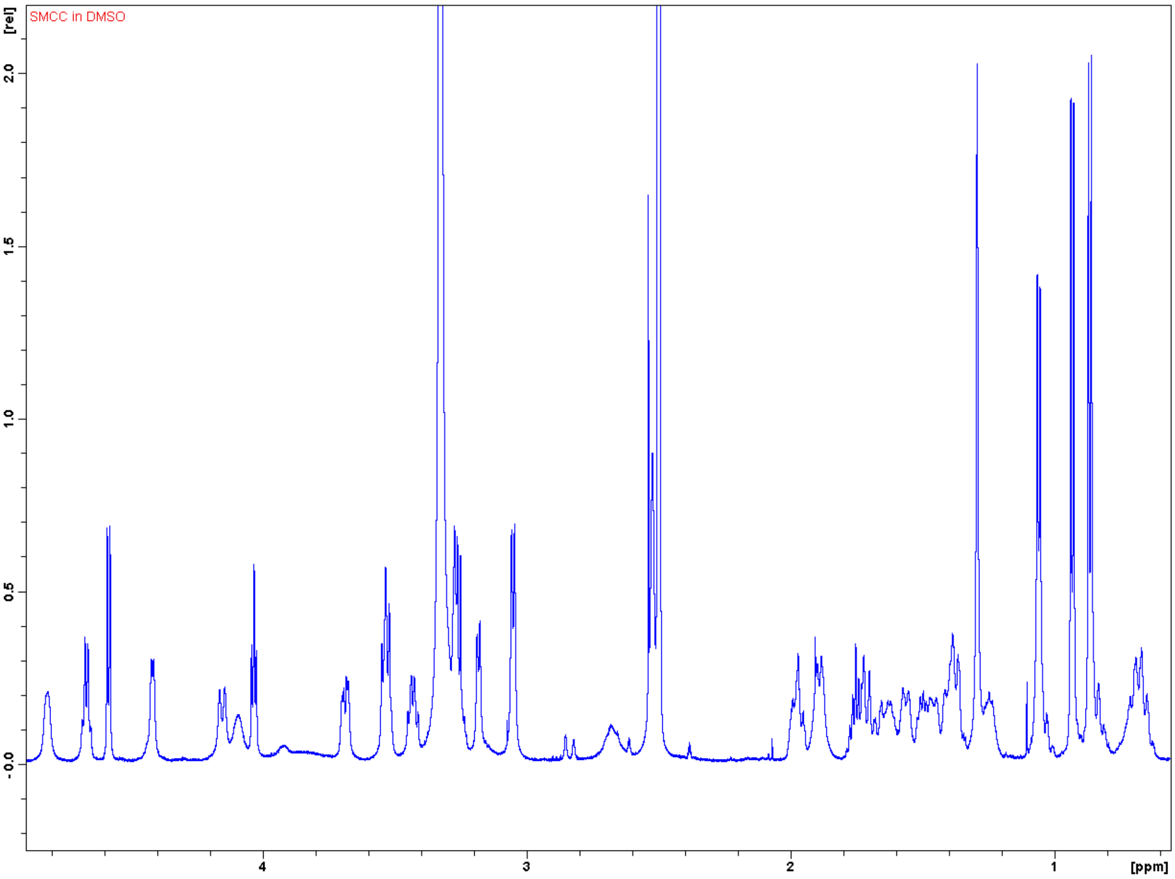

Figure S8. Upfield region of the ^{1}H NMR spectrum of SMCC.

Intermediate product of VAN:PEG-derivatives: FU002ySMCC

^{1}H (600 MHz, DMSO), major (M) and minor (m) species (2:1): 13.02, v br s (m); 12.95, v br s (M); 10.00, s (m); 9.67, s (M); 9.41, s (M); 9.39, s (m); 9.20, s (M+m); 9.07, v br s (M+m); 8.98, br s (M+m); 8.79, br d, *J* ~ 4.3 (m); 8.75, br s (M); 8.69, br s (m); 8.58, br s (M+m); 8.32, br d, *J* ~ 5.4 (M); 8.18–7.96, 5.6H, m; 7.86, v br s (M+m); 7.80, s (M+m); 7.72–7.58, 9H, m; 7.56, d, *J* = 8.7 (m); 7.54, d, *J* = 8.3 (M); 7.47, d, *J* = 8.6 (M); 7.34, d, *J* = 8.4 (m); 7.34, d, *J* = 8.3 (M); 7.30–7.25, br m (M); 7.22, v br s (m); 7.20, d, *J* = 8.9 (M); 7.18, v br s (m); 7.09, v br s (m); 7.01, AA' part of AA'XX' m, $J_L + J_S$ = 8.2 (M+m); 7.01–7.00, 2.8H, m; 6.82, d_{AB}, *J* = 8.5 (M); 6.78, v br s (m); 6.76, d_{AB}, *J* = 8.5 (M); 6.72, d_{AB}, *J* = 8.6 (m); 6.70, d, *J* = 2.0 (m); 6.68, d_{AB}, *J* = 1.6 (M); 6.63, AA' part of AA'XX' m, $J_L + J_S$ = 8.4 (M+m); 6.56, d, *J* = 2.1 (m); 6.42, d, *J* = 1.4 (M); 6.02–5.95, 2H, m (M+m); 5.79, d, *J* ~ 9.8 (m); 5.78, d, *J* = 7.9 (M); 5.66, br s (m); 5.62, br s (M); 5.49–5.45, ol m (M+m); 5.36, d, *J* = 4.5 (M+m); 5.28–5.22, 2.2H, m; 5.20–5.15, 1.8H, m; 5.15–5.07, 2.5H, m; 4.91, v br s (M); 4.87, v br s (m); 4.68, ~qt, *J* = 6.5 (M+m); 4.57, d, *J* = 6.0 (m); 4.50, d, *J* = 5.2 (M); 4.56, v br s (M); 4.43–4.30, 1.4H, m; 4.29–4.12, 8.7H, m; 4.10–4.01, 2.2H, m; 4.01–3.93, m (M+m); 3.69, br d, *J* = 10.2 (M+m); 3.57–3.50, 1.9H, m; 3.46–3.41, 2H, m (M+m); 3.27, 2H, v br s (M+m); 3.22, d, *J* = 6.9 (M+2m); 3.17, br d, *J* = 5.9 (M); 3.12–2.98, 15.5H, m; 2.89, dd, *J* = 12.9, 9.3 (m); 2.82, dd, *J* = 13.6, 4.8 (M); 2.68, dd, *J* = 13.2, 9.7 (M); 2.65–2.54, 3.8H, m; 2.19–2.11, m (M); 2.11–2.02, 3.3H, m; 1.94–1.86, m (M+m); 1.78–1.25, 42.4H, m; 1.29, 3H, s (M); 1.29, 3H, s (m); 1.18, br qt, *J* = 12.1 (M); 1.13–1.01, 2.3H, m; 1.07, 3H, d, *J* = 6.1 (M+m); 0.94–0.79, 2.7H, m; 0.92, 3H, d, *J* = 5.4 (m); 0.91, 3H, d, *J* = 5.9 (M); 0.87, 3H, d, *J* ? (m); 0.86, 3H, d, *J* = 6.5 (M); 0.79–0.68, m (M+m).

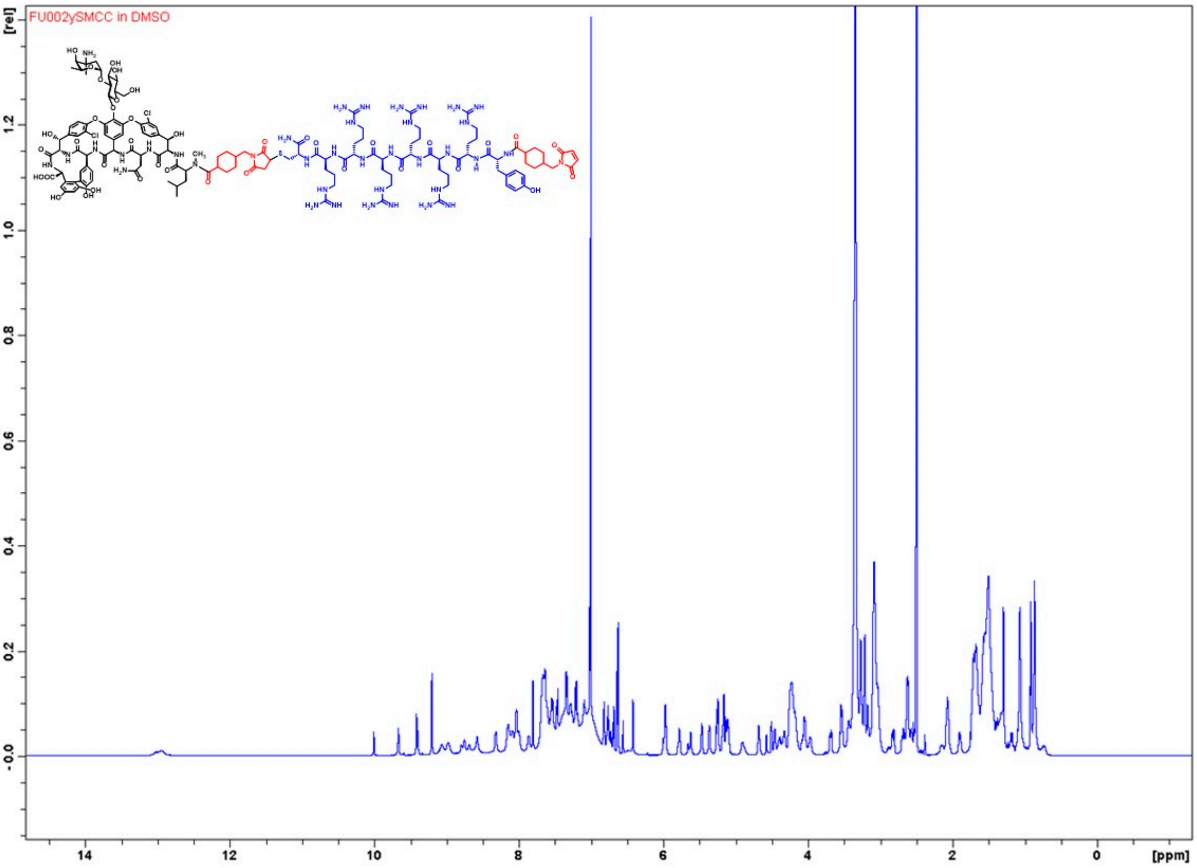

Figure S9. ^{1}H NMR spectrum and structure of FU002ySMCC.

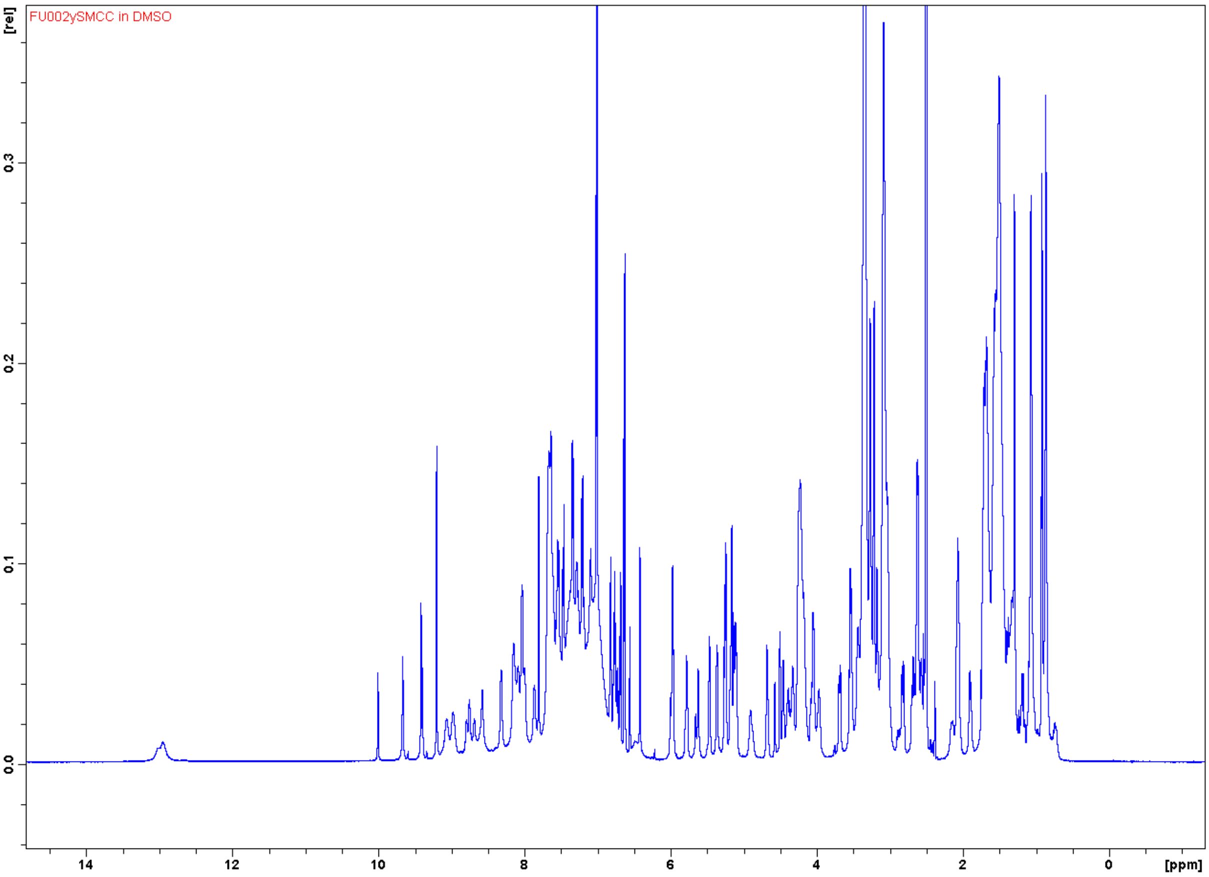

Figure S10. ^{1}H NMR spectrum of FU002ySMCC scaled up.

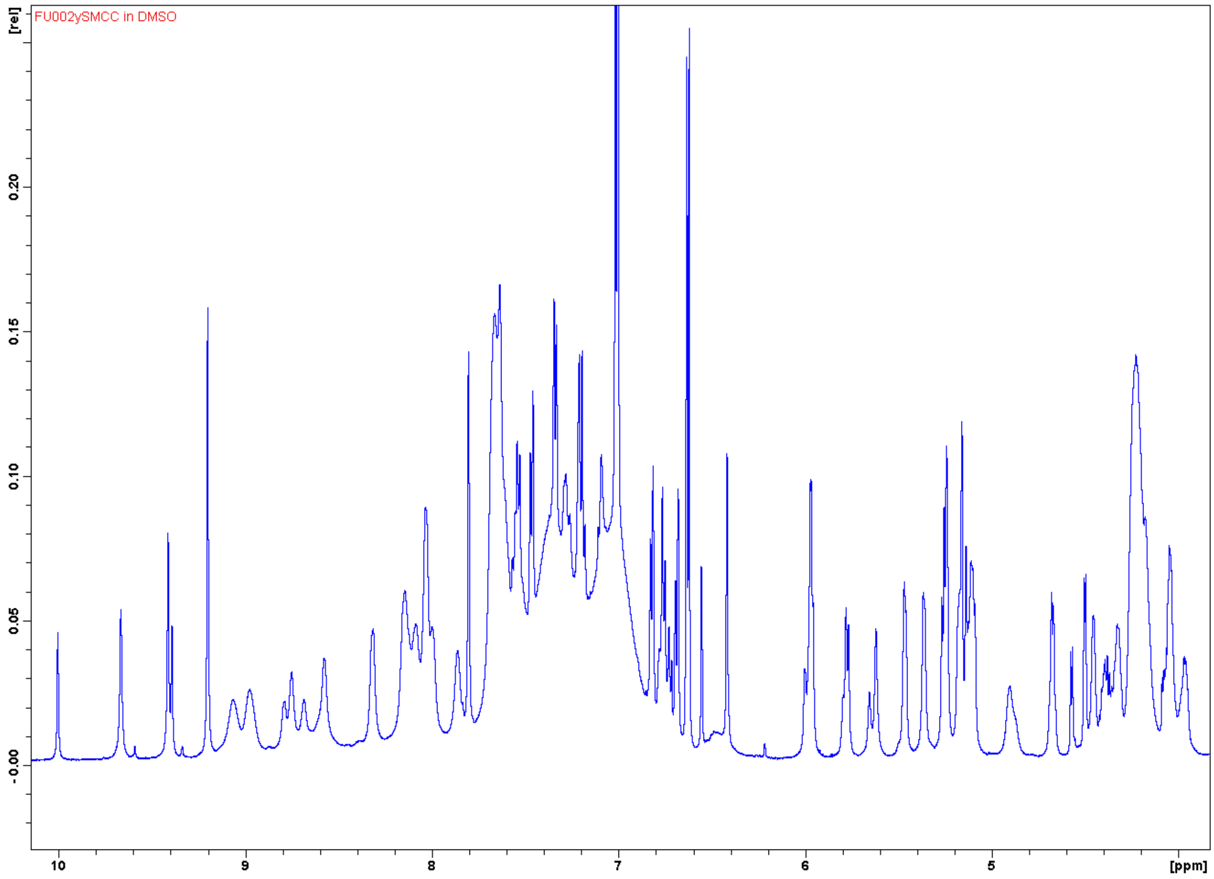

Figure S11. Downfield region of the ^{1}H NMR spectrum of FU002ySMCC.

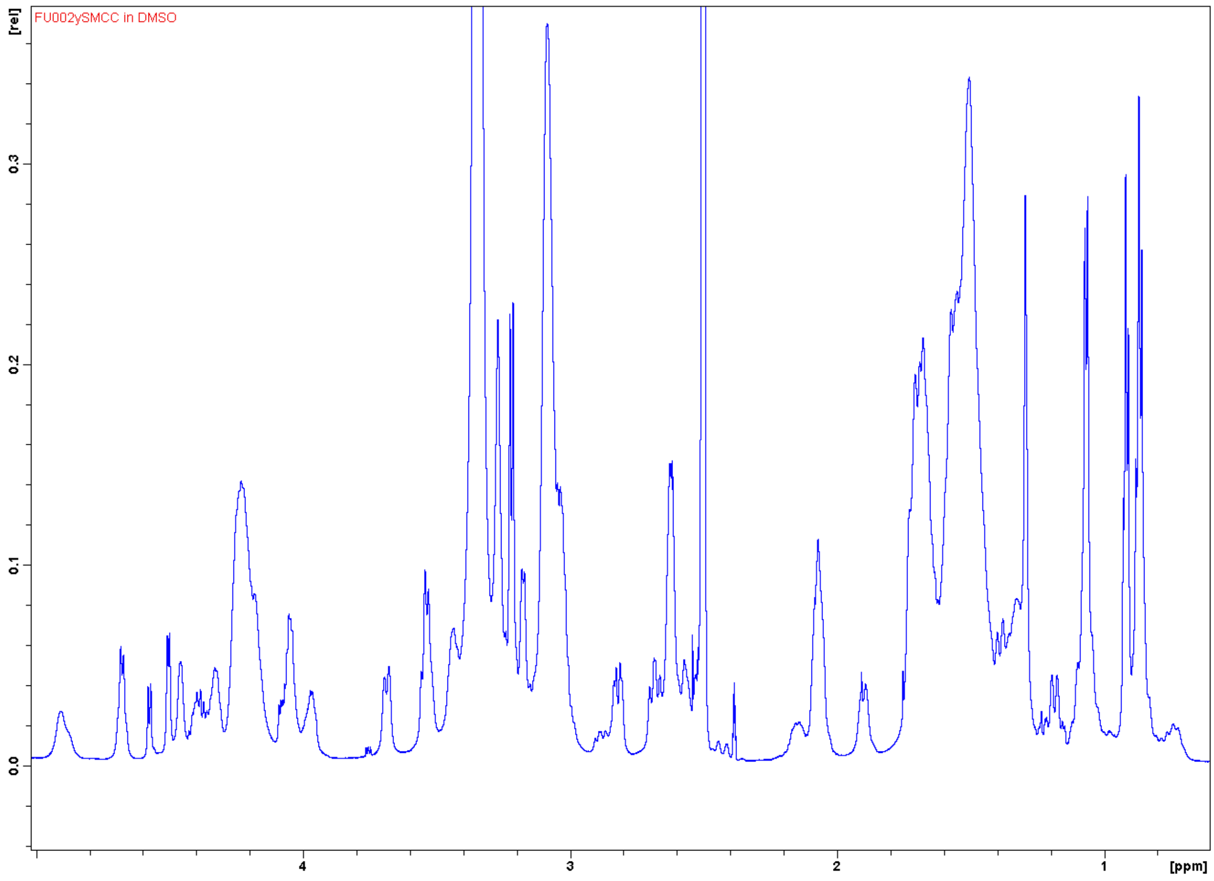

Figure S12. Upfield region of the ^{1}H NMR spectrum of FU002ySMCC.

Antimicrobial activity

Table S1. Minimum inhibitory concentration against B. subtilis DSM10. All tested conjugates showed high antimicrobial activity. Shown is the median of at least three independent measurements (range is shown in brackets).

**Compound**	**Minimum Inhibitory Concentration against *B. subtilis* DSM10 [mg/L]**
VAN1	1 (0.5–1)
VAN2	2 (1–4)
VAN3	<0.125
Vancomycin	0.25 (0.125–0.5)

Hemolysis studies

Table S2. Hemolysis study of VAN1. No significant hemolytic activity could be observed (n = 3).

**Concentration [mg/L]**	**VAN1 [% hemolysis]**
120.00	1.44 ± 1.02
60.00	0.56 ± 0.52
30.00	0.33 ± 0.23
15.00	0.14 ± 0.07
7.50	0.05 ± 0.13
3.75	0.02 ± 0.16
1.88	0.09 ± 0.15
0.94	−0.02 ± 0.10
0.47	−0.07 ± 0.16
0.23	0-01 ± 0.21

Table S3. Hemolysis study of VAN2, VAN3 and VAN:PEG1. No significant hemolytic activity could be observed (n = 3).

**Concentration [mg/L]**	**VAN2 [% hemolysis]**	**VAN3 [% hemolysis]**	**VAN:PEG1 [% hemolysis]**
16.00	0.60 ± 0.78	0.28 ± 0.37	0.31 ± 0.40
8.00	0.39 ± 0.44	−0.06 ± 0.31	0.34 ± 0.28
4.00	0.21 ± 0.20	−0.11 ± 0.36	−0.01 ± 0.25
2.00	0.20 ± 0.16	−0.09 ± 0.31	−0.04 ± 0.22
1.00	0.19 ± 0.23	0.13 ± 0.17	0.40 ± 0.48
0.50	0.10 ± 0.16	0.14 ± 0.16	0.27 ± 0.24
0.25	0.00 ± 0.22	0.06 ± 0.16	−0.05 ± 0.19
0.13	−0.02 ± 0.22	−0.20 ± 0.46	−0.15 ± 0.26
0.06	−0.07 ± 0.24	−0.23 ± 0.44	0.01 ± 0.21
0.03	−0.15 ± 0.23	−0.28 ± 0.46	−0.02 ± 0.17

References

S1. Pearce, C.M.; Williams, D.H. Complete assignment of the ^{13}C NMR spectrum of vancomycin. *J. Chem. Soc., Perkin Trans.* **1995**, 2, 153-157.
